# A dialogical conception of *Habitus*: allowing human freedom and restoring the social basis of learning

**DOI:** 10.3389/fnhum.2014.00432

**Published:** 2014-06-17

**Authors:** Kleio Akrivou, Lorenzo Todorow Di San Giorgio

**Affiliations:** Leadership, Organisations and Behaviour, Henley Business School, University of ReadingReading, UK

**Keywords:** consciousness, conversation, dialogue, habitus, human development, processual self, learning, neuroscience

## Bourdieu's construct of habitus and critique

The current sociological understanding of habitus expressed in the work of Pierre Bourdieu as its key academic construct emphasizes the importance of habits for human action. Bourdieu understands human behavior as fundamentally cultural; rejecting behaviorist view of cognition and action as related to a stimulus—response chains, the French sociologist posits instead that human action emanates from internalized habits (Swartz, 2002). Bourdieu, utilizes the concepts of field and *habitus*—the latin word for habit- (1990/1977), to critically analyze the microsphere of society and economy elevated as habituated human action (Bourdieu, 2005).

Bourdieu understands action as habituated, related to the unconscious reproduction of external social fields. This allows *habitus* to be this foundational concept it is in Bourdieu's reflexive sociology, examining societies as socio-economic relations and classes, rather than actors. His emphasis on the social context's influence on human habits being internalized relatively unconsciously is important, as it looks to action-interaction sequences as habituated, opposing behaviorist (Swartz, 2002) models of human action as simple stimulus-response chains, or objectivist accounts. Bourdieu's *habitus* emphasizes two things relevant to human action: (a) opposing consciousness to habit(us), which continues the modern sociological tradition from Weber (Camic, 1986) and, (b) the socially learnt (externally located) nature of habit(us). A habit is viewed as an unconscious principle of action, a deeply internalized set of dispositions, schemas and ways of knowing (Swartz, 2002) which locate habits in a cultural, economic, or social field.

In our opinion, the main problem in Bourdieu's view of *habitus* is that it largely accounts for human action being reproductive of an existing field, rather than transformative. Because the Bourdieuian *habitus* is theorized as an adopted “thrown way of being” in the world (Akrivou and Bradbury-Huang, 2014 forthcoming) it blocks human freedom with social bonds, as action is posited to emerge directly from the internationalization of norms of relational exchange in the outside field(s) of practice.

Our critique regarding Bourdieu's (quintessential) sociological *habitus* is that its conception explains processes accounting for human behavior regulation to carry forward existing conventions and rules, reproductive of existing social bonds; it is less mindful to processes of critical questioning or transformation of an existing status quo and the role of human cognition in generating action which can interrupt and interrogate the field.

To explain our critique further, even when Bourdieu accepts that actors can generate new action, he understands the “new” as habits from earlier socialization. Our main concern with Bourdieu's view is that looking to *habitus* as outside introjection means that individual *habitus* is in the best case “an active residue of (one's) past” (Swartz, 2002, p. 63S) which is Bourdieu's view! But we wish to critique this as it theoretically misses to account for the possibility for human freedom, which can be appreciated by reference to other understandings of *habitus* (Aristotle's for example).

## A dialogical conception of habitus, supported by neuroscience, and psychological theory on human development

We believe that our critique of Bourdieu's *habitus* enables us to argue, that, in the frame of a dialogical conception *habitus* can be compatible with the social basis of human freedom and learning. Bourdieu's *habitus* defines it as site of replication of social bonds and boundaries, unless we revise his conception with a generative less deterministic structure, which protects the possibility of new habits emerging from agency. A dialogical conception understands human agency to be simultaneously part of a field of practice (and earlier socialization), and open to a gradual co-creation of novel action. The latter, emerging from an intentional conversational engagement practice between acting agents, can release a *new experience* of in-betweeness cognition, as dialog is a reciprocal “mode of communication that builds mutuality through awareness of others and as an instance of unfolding interaction” (Eisenberg and Goodall, 1993; Bohm, 1996; Putnam and Fairhurst, 2001, p. 116; Ballantyne, 2004). A dialogical conception of habitus can be compatible with the social basis of human freedom and learning, and core philosophical theories illustrate how dialog and conversation can gradually catalyze new *habitus*.

Buber's and Gadamer's **ethics of dialog** are relevant to our argument. Buber understands human freedom to emerge from locating oneself ethically in genuine relationships of a reciprocal “world of relation” (Buber, 1970, p. 56), with another fellow human. For Buber, the difference between “I-Thou” and “I-It” is not in the nature of the object to which we relate, but in the binding relationship itself (Levinas, 1989; Buber, 2002). Responding ethically to “Thou” replaces a passive response habit, an unreflective reproduction of external sets of relations and previously learnt dispositions. To keep dialogical ways of responding, one must engage in shared reflection to how to mindfully develop a “quality of genuine relationship in which partners are mutually unique as whole… this deep bonding is contained neither in one, nor the other, nor in the sum of both- but becomes really present between them” (Kramer, 2003, p. 15).

Gadamer also sees dialog as the *process* fostering a gradual mutual development of a shared gradually binding quality of relatedness. Gadamer notes that “to conduct a conversation means to allow oneself to … be caught up in something larger, which is neither subjective, nor objective, neither totally relative or fixed, … but … a structural unity … being conducted by the subject matter to which the partners in the dialog are oriented” (Gadamer, 1965, p. 367). Conversation partners gradually become less preoccupied with safe habits and engage in reaching a shared truth (White, 1994) with regard to how to proceed in a shared quest for *die Sache -or subject matter of inquiry* (Gadamer and Lawrence, 1982; Kelly, 1988; Gadamer, 1989, p. 383). The relationship becoming gradually a binding *“play of persons,”* the bond being conversation itself (White, 1994).

We argue that, a dialogical conception of habitus releases human freedom. Based on the previous analysis, engagement in dialogic habitus gradually forms a semi-autonomous zone (Akrivou and Bradbury-Huang, 2014) of action, which can generate new ways of knowing, while it also converses with habitus of the outside field of practice. In this argument, the idea of human freedom is meaningless outside the conversational practice; instead the necessity challenges of dialogic habitus requires to transcend the conventional assumption of independently autonomous rational agency to engage in the conversational “structural unity” (Gadamer, 1965; Akrivou and Bradbury-Huang, 2014) with a specific other fellow human. Developing the argument here the necessity challenges of dialogic ethics as a way to help address the critique of Bourdieu's *habitus*.

This argument can be supported by advances in neuroscience and psychological theory. It may seem new to many Westerners the idea of dyadic conversational structures (an I-thou structural unity) being the locus of consciousness as the sole or primary arbiters of social action; rather than each individual solitary independent autonomous rational processing (Akrivou and Bradbury-Huang, 2014). This supports revising the conception of human brain and cognitive processing, opposing a human brain operating via top-down predictions about sensory inputs and fully predicts the sensory information being received (Benacer and Murillo, 2012). Conversation is dynamically releasing new shared cognition pathways as one gradually learns to listen, feel, respond and engage in thoughtful responsiveness to a specific other actor.

The implication of such view of the locus of social action means that each human being bears the possibility of freedom (and the responsibility) to reflect what one brings forth in the world of relations and how. Once a dialogical conception is present in a given social field of action between inter-dependent agents *habitus* can be compatible with the social basis of human freedom and learning. A dialogical habitus opposes many Western philosophical theories emphasis on detached, autonomous scientific rationality. It instead supports neuroscience research that we are endowed with a brain adapted (Gazzaniga et al., 2002) to parcel out reality as separable units of an ever-changing flow of experience. We learn that solving self occurs mainly in the prefrontal cortex, with the emotional self-arising from the amygdala (Lewis and Todd, 2007). Any momentarily active aspects of the self-engage a fraction of the brain's networks (Gusnard et al., 2001; Legrand and Ruby, 2009). Contrary to our deeply and psychosomatically held belief in ourselves as “distinct individuals,” many personal aspects happen automatically such as our heart beating. “In effect” summarizes Hanson, “subjectivity arises from the inherent distinction between this body and that world (2009, p. 210).” Indeed, Koch and Tsuchiya (2006) have found that diminishing habitual self-consciousness yields more positive results for the performer. Western neuroscience is pointing to the tantalizing fact that subjectivity is a way to structure experience but is not necessarily linked to individual persona (Hanson, 2009, p. 212).

Neuroscience is also supported by insights from human learning (Kolb, 1984; Maturana and Varela, 1987; Varela et al., 1991) and human development theory (Dewey, 1929; Werner, 1948; Harvey et al., 1961; Rogers, 1961; Schroder et al., 1967; Loevinger, 1976; Kohlberg and Ryncarz, 1990) on superior human cognitive moral maturity capacity. This is seen grounded in human freedom to choose both to be moral and **how** to engage in qualitative ways; “how” refers to a certain quality of cognitive processing which transcends subjectivity and engages in fluid, mutual inter-subjective ways of knowing (Rogers, 1961; Kegan, 1994). This quality of experiencing cognition is possible once a person freely “gives up” the safety of one's autonomous self-authorship on the basis of solitary reason—an ideal of conventional moral maturity based on the (Piaget, 1962) theory of development toward formal operations relying on abstract knowing (Flavell, 1963; Loevinger, 1976).

Rather than a mechanical (mono-logical), crystallized cognitive map already stored in the brain in the form of abstract schemas, enabling a purely adaptive cognitive processing on the basis of habituated knowing how to respond to a set of outside stimuli, being in conversation intervenes to change the very way a previously taken for granted form of action can be experienced and dynamically practiced anew. Transcending its very reliance on formal operational thinking, the **processual self** emerges from within a dialogical habitus experiencing, an organic way of being complete (integrated) *in situ* from within the process of narrative relational responsiveness (Akrivou, 2008), whereby one engages in the experience of relating genuinely with another human being as ground rather than a figure (James, 1979; Kohlberg and Ryncarz, 1990; Gendlin, 1997). This gradually develops a diverse set of the brain's cognitive pathways, as Bradbury- illustrates (in press) we learn “over time, to skillfully be with experience.”

The emergence of previously unthought degrees of freedom generates novel action and social learning from within conversational fields itself rather than previously known habituated response schemas (Akrivou and Bradbury-Huang, 2014). This idea can be illustrated by the language of co-emergence in enactivist theories of human learning (Maturana and Varela, 1987; Varela et al., 1991). A dialogic process of narrative consciousness replaces cultural tools, rules, conventions and language as mechanisms for action regulation. It is instead a dynamic view of human cognition, a socially responsive brain which intentionally self regulates itself to context and other human beings own responses (Lewis and Todd, 2007). This conception of *habitus* generates meaningful novel action, binding one's own conscious attention and other actors' causal intervention responses in the process of shared conversational learning (Baker et al., 2002).

In conclusion, in the frame of a dialogical conception supported by psychological and neuroscientific findings, *habitus* can be compatible with the social basis of human freedom and learning. A dialogical conception of *habitus* may allow for habitus counter-intuitive to Bourdieu (Akrivou and Bradbury-Huang, 2014) which can be compatible with the social basis of human freedom and learning. It is closer to Aristotle's idea that rational agents (ought to) remain conscious of which habits to embrace and an active role of human agents being a consequence of engaging with virtuous habits. Perhaps, our argument helps bring Bourdieu's habitus closer to Aristotle's inquiry on the significance on human intentional action for a social world capable for virtue.

### Conflict of interest statement

The authors declare that the research was conducted in the absence of any commercial or financial relationships that could be construed as a potential conflict of interest.
